# Death of Dr. James McFadden Gaston

**Published:** 1903-12

**Authors:** 


### *EDITORIAL NOTES AND COMMENTS.*

The Business office of THE JOURNAL-RECORD is No. 1007-1008 Century Building.

The Editorial office is 318-319-320 The Prudential.

Address all Business communications to Dr. M. B. Hutchins, Mgr.

Make remittances payable to THE ATLANTA JOURNAL-RECORD OF MEDICINE.

On matters pertaining to the Editorial and Original Communications address Dr. Bernard Wolff, Atlanta.

Reprints of original articles will be furnished at cost price. Requests for the same should always be made *on the manuscript*.

We will present, post-paid, on request, to each contributor of an original article, twenty (20) marked copies of THE JOURNAL-RECORD containing such article.

### DEATH OF DR. JAMES McFADDEN GASTON.

DR. JAMES McFADDEN GASTON died on Sunday morning November 15.

With the death of Dr. Gaston the city and the State loses one of its most illustrious physicians. Dr. Gaston's reputation was not circumscribed by State limits, because his name was known and respected in the medical literature of every country. He had the spirit of investigation and the courage of his convictions. He worked with a fixed purpose in view and was an indefatigable seeker after truth. As a man he enjoyed the esteem and affection of his colleagues to a notable degree. He was singularly kind and considerate of the younger practitioners, and before the infirmities of age crippled his usefulness his counsel and advice were much sought after by the younger men in the profession, while his ripe experience and sound judgment commended him as a favored consultant among the older men of his craft. He was the soul of kindness and professional integrity, and though so frequently consulted he would have scorned those methods of self-aggrandizement and insinuation for the usurpation of another's rights—a fault said to be prevalent in the profession of medicine. He was a friend, faithful and just. He lived out a life of unselfish work and ceaseless activity, and though enfeebled by age with its attendant debility and weakness, his interest in his profession was unflagging, and when the call came he yielded up his spirit to his Maker whom he had served with swerveless faith so long, for he knew in whom he had trusted.

Dr. Gaston, the son of Dr. John Brown and Polly (Buford) Gaston, and grandson of Joseph Gaston, was born December 27, 1824, near Chester, S.C. He attended the common schools of his native county, and obtained an academic education at Russell Place in Kershaw District. At the age of sixteen he entered the South Carolina College, Columbia, and was graduated A.B. in December, 1843; commenced the study of medicine in 1843 at his home in Chester, under the direction of his father, Dr. John B. Gaston; attended one course of lectures at the University of Pennsylvania, Medical Department, and one course at the Medical College of the State of South Carolina, receiving from this institution the degree of M.D., March 6, 1846. He immediately entered upon the practice of medicine in Chester District, S. C., in partnership with his father, which relation was continued until the fall of 1852, when he removed to Columbia, S. C. At the opening of the civil war, Dr. Gaston enlisted in the Columbia Grays, and entered service at Morris Island, where he was appointed chief surgeon of the South Carolina forces under the command of Brigadier-General M. L. Bonham. Surgeon Gaston accompanied General Bonham to Richmond, Va., and when the troops were removed to Manassas, he was assigned as medical director of the department under Brigadier-General G. T. Beauregard. After the first battle of Manassas, Dr. Gaston, at his own request, was transferred by General Beauregard to the Third Brigade South Carolina Volunteers, under Brigadier-General R. H. Anderson, until this officer wa s appointed major-general. Dr. Gaston was then promoted to chief surgeon of his division, and participated in Virginia and Pennsylvania campaigns. By special order of the surgeon-general Dr. Gaston went to the relief of the wounded after the battle of Chickamauga, and assisted Dr. S. H. Stout, medical director of hospitals, in the secondary operations at Marietta. An application was made by Surgeon Stout for the transfer of Surgeon Gaston to his department, but he was ordered, instead, to report to the medical director of hospitals in General Beauregard's division, and was sent to establish a general hospital at Fort Gaines, Georgia. He was subsequently in charge of a general hospital at Fort Valley, where he remained on duty until the close of the war.

After the cessation of hostilities in 1865, Dr. Gaston went to Brazil, where he attended the lectures of the Imperial Academy of Medicine, and in 1873 received the *ad eundem* degree, entitling him to practice medicine in that country. He was offered the position of consulting surgeon of the military medical staff of Brazil, but declined. After removing with his family to the Province of St. Paulo, in 1867, Dr. Gaston practiced his profession six years in the interior towns. In 1874 he removed to the city of Campinas, Brazil, and practiced medicine there until his return to the United States in 1883, since which time Atlanta has been his permanent residence. Soon after settling in Atlanta he opened a surgical infirmary in connection with his surgical practice, and in 1884 was elected professor of the principles and practice of medicine in the Southern Medical College, Atlanta, to which he has since devoted his best energies.

Dr. Gaston has made experiments upon dogs for the formation of a communication between the gall-bladder and the duodenum, or upper portion of the small intestine. He also introduced the abdominal spring pessary, upon which the McIntosh instrument has been extensively employed in recent years. Of his medical papers, those treating of carbuncle, erysipelas, yellow-fever inoculation, appendicitis, ovariotomy, traumatism of the chest, and surgery of the gall-bladder and ducts have received the greatest consideration.

Dr. Gaston is a member of the American Medical Association, chairman of its surgical section, 1891–92; of the Southern Surgical and Gynecological Association, president in 1892; of the American Academy of Medicine, president in 1895; of the Medical Association of Georgia; and of the American Surgical Association.

Married, November 2, 1852, Miss Sue G. Brumby, daughter of Professor R. T. Brumby, of the University of South Carolina, Columbia. Of their ten children, the following are living: Mrs. Mary Buford Gresham, Mrs. Keziah Brevard Kolb, Mrs. Nanny Thornwell Blackford, Mrs. Kate Shaw, Mrs. Susan Eloise Gay, and Dr. James McFadden Gaston, Jr.

